# Investigation of the polarization state of dual APPLE-II undulators

**DOI:** 10.1107/S1600577515021645

**Published:** 2016-01-01

**Authors:** Matthew Hand, Hongchang Wang, Sarnjeet S. Dhesi, Kawal Sawhney

**Affiliations:** a Diamond Light Source, Harwell Science and Innovation Campus, Didcot, OX11 0DE, UK

**Keywords:** polarization, multilayers, undulators

## Abstract

The use of an APPLE II undulator is extremely important for providing a high-brilliance X-ray beam with the capability to switch between various photon beam polarization states. A high-precision soft X-ray polarimeter has been used to systematically investigate the polarization characteristics of the two helical APPLE II undulators installed on beamline I06 at Diamond Light Source. A simple data acquisition and processing procedure has been developed to determine the Stokes polarization parameters for light polarized at arbitrary linear angles emitted from a single undulator, and for circularly polarized light emitted from both undulators in conjunction with a single-period undulator phasing unit. The purity of linear polarization is found to deteriorate as the polarization angle moves away from the horizontal and vertical modes. Importantly, a negative correlation between the degree of circular polarization and the photon flux has been found when the phasing unit is used.

## Abstract 2

Complete polarization analysis of the photon beam produced by a dual APPLE-II undulator configuration using a multilayer-based soft X-ray polarimeter is given.

## 1. Introduction   

Next-generation synchrotron radiation sources are capable of delivering unprecedented brilliant soft X-ray beams with adjustable polarization state thanks to the modern undulators (Sasaki, 1994 ▸; Hwang & Yeh, 1999 ▸; Weiss *et al.*, 2001 ▸). Nowadays, many experimental methods utilizing polarized synchrotron radiation – for example, X-ray magnetic linear/circular dichroism (Stöhr *et al.*, 1998 ▸; Ghidini *et al.*, 2015 ▸) and X-ray magnetic scattering (Dürr *et al.*, 1999 ▸) – are heavily reliant on a photon beam with a pure and well defined polarization. The use of a variable polarization undulator provides great flexibility for meeting the demands of such experiments, but factors such as magnetic field inhomogeneity, magnet misalignment and beamline optics can influence the final polarization state of the beam at the sample position. Therefore, accurate knowledge of the polarization state of the photon beam is crucial in order to be confident in the results obtained from such measurements. Nowadays, a single long undulator is well established to deliver the high flux required for many experiments. However, it is usually time consuming to switch the polarization state (such as from left-handed circular to right-handed circular) for a single undulator by changing both the gap and row phase. It is possible to overcome this limitation by replacing a long undulator with two shorter undulators (Bahrdt *et al.*, 2001 ▸; Quitmann *et al.*, 2001 ▸; Weiss *et al.*, 2001 ▸), so prompt polarization state switching can be realised by widely opening one undulator gap (for example, left circular for one undulator and right circular for the other). In addition, the total delivered flux from two short undulators is comparable with that from a single long undulator with the same length when using an adjustable phasing unit. Whereas each undulator might be well calibrated individually, the final polarization performance of dual undulators could vary due to the mismatch of the phasing unit. Hence, it is essential to perform *in situ* polarization measurements to understand the undulator and beamline performance. Although complete polarization analysis for a single undulator has been well characterized in the soft X-ray region (Schäfers *et al.*, 1999 ▸; Wang *et al.*, 2007 ▸, 2012 ▸; MacDonald *et al.*, 2009 ▸), limited efforts have been made to investigate the polarization state of dual undulators so far. Unlike polarization measurement in the vacuum ultraviolet region (Nahon & Alcaraz, 2004 ▸; Bahrdt *et al.*, 2010 ▸), precise diagnosis of the polarization state in the soft X-ray region is particularly challenging owing to the lack of polarizing elements and stringent requirements for the alignment of the polarimeter (MacDonald *et al.*, 2009 ▸; Wang *et al.*, 2011 ▸).

Here, a high-precision polarimeter has been employed to systematically characterize the polarization state of dual APPLE II undulators with a phasing unit. Importantly, we have shown the effect from the phasing unit on the polarization state when the two undulators were phased together. Such precise polarization measurement will provide valuable input for polarization-related experiments with such dual undulators.

## 2. Experimental details   

**Figure 1:**
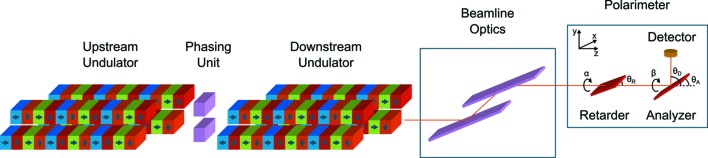
Schematic of the undulator configuration at beamline I06 with polarimeter. The retarder/analyser tilt angles (θ_R_/θ_A_) are measured from the optical axis to the corresponding element surface and the retarder/analyser azimuthal angles (α/β) are measured from the vertical *y* axis.

The complete polarization analysis of dual undulators was carried out on beamline I06 at Diamond Light Source (Dhesi *et al.*, 2010 ▸). A schematic of the experimental set-up is shown in Fig. 1 ▸. Two identical helical undulators (APPLE II-type) were systematically investigated with a high precision soft X-ray polarimeter (Wang *et al.*, 2011 ▸). Each APPLE II undulator is equipped with two movable magnet arrays and a variable gap which provides polarization selection and high flux over the core energy range (70–2100 eV). It is possible to select any of the following polarization states: linear horizontal (LH), linear vertical (LV), linear at arbitrary angle, left-handed circular (LC), right-handed circular (RC) or elliptical. Each undulator is 2.11 m in length and has 33 periods with period length λ_u_ = 64 mm. The minimum gap between the magnet rows is 15 mm and photons can be produced in the 70–1300 eV energy range on the first harmonic in linear horizontal mode (circularly polarized photons are not available below 106 eV and LV photons can only be produced above 130 eV). The energy range of the beamline is extended up to 2100 eV by utilizing the third undulator harmonic. The two undulators are configured completely independently so if each device is set for one of the two polarization states required (for example, left-handed circular on one undulator and right-handed circular on the other) then fast switching of the polarization state is possible (limited here by the time required to completely open the undulator gap ∼3–4 s). Additionally, a single-period undulator – or phasing unit (PU) – located between the two insertion devices enables the outputs from each undulator to be phase shifted with respect to one another, increasing the total photon flux available at the sample. A beam position monitor is also located between the two devices.

The measurements presented here were carried out at the I06 branch line; the main line is permanently occupied by a photoemission electron microscope end-station. A single collimated plane grating monochromator (Follath & Senf, 1997 ▸) serves both branches and contains gratings with line densities of 150 lines mm^−1^ and 400 lines mm^−1^ (used here) optimized for use at low and high energies, respectively. A 1200 lines mm^−1^ grating for high-energy resolution measurements is also fitted. Gold coatings are used for each of the gratings and all the beamline mirror optics.

The high-precision Diamond polarimeter contains a transmission multilayer phase retarder and a reflection multilayer analyser (Wang *et al.*, 2011 ▸). Both the retarder and analyzer can rotate independently about the optical axis of the beam (angles α and β, respectively) and a photodiode is used to collect the transmitted photon flux. The polarimeter chamber is mounted on a hexapod stage which provides six degrees of freedom for alignment. Externally mounted fiducial markers allow for coarse positioning and pre-alignment of the polarimeter optical axis to the X-ray beam using a laser tracker. Fine alignment of pitch and yaw is achieved using X-rays by scanning the analyzer tilt angle θ_A_ across the Brewster angle with a fixed detector position (θ_D_ = 2θ_A_) at different opposing β angles (0/180° for yaw adjustment and 90/270° for pitch adjustment). Perfect alignment is achieved when the reflection peak centres are coincident, so they are fitted to determine their angular separation and an adjustment is made to the corresponding motion of half this value. This procedure is automated and can be repeated several times in order to position the polarimeter optical axis within 50 µrad of the beam axis.

**Figure 2:**
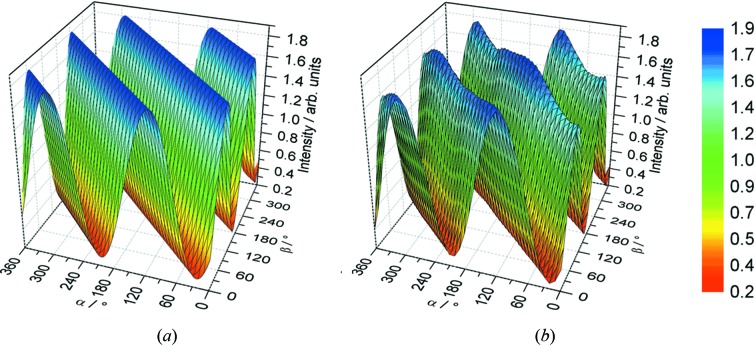
Three-dimensional map of left circular polarized light intensity at 375 eV from (*a*) theoretical calculation and (*b*) measurements using the Diamond polarimeter. The similarity of the two surfaces is indicative of the precise alignment of the optics and their axes of rotation.

In each polarization measurement one of the axes α or β is designated as the primary rotation. At each angular point of the other (secondary) rotation, data are acquired for a complete rotation of the primary axis. A measurement for rotation of both axes at ‘high’ resolution (say every 10°) provides a complete map of the detected light intensity, which is characteristic of the photon polarization state. Fig.2 ▸ demonstrates the agreement with theoretical calculation (Wang *et al.*, 2012 ▸) for LC light. The only discrepancy is a variation in observed peak intensity which indicates the presence of a linear component in the otherwise circularly polarized photon beam.

For reliable fitting it is sufficient to measure points at high resolution on the primary axis only; a much larger angular spacing (*e.g.* 45°) may be used for the secondary rotation, significantly reducing the total number of data points to be acquired. In addition, the mechanics of α and β limit them to a single 0–360° rotation before they must be driven in the opposite direction. Therefore, alternate scans of the primary rotation occur in the reverse direction, *i.e.* 360–0°, so the time spent travelling back to 0° between scans is not wasted. This optimization of the data acquisition procedure allows a data set for complete polarization analysis to be captured in approximately 25 min.

**Figure 3:**
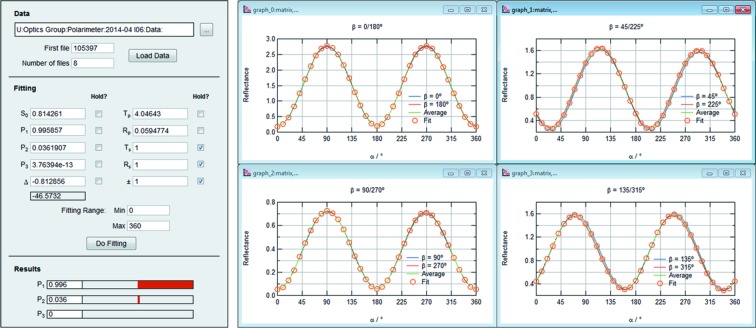
*Igor Pro* GUI for selection of polarimeter data, input and display of fitting variables, and presentation of best-fit Stokes–Poincaré polarization parameters. A complete data set is shown from a measurement of LH light – the data from opposing β angles are averaged and used for the fitting procedure.

The expression used in the previously mentioned calculation is used as the basis for a least-squares fitting routine, implemented by the author in *Igor Pro 6.32A*, which contains seven free parameters: the three Stokes–Poincaré parameters (*P*_1_, *P*_2_ and *P*_3_), the retarder phase shift (Δ), the phase retarder ratio of *p* to *s* transmission (*T*_*p*_/*T*_*s*_), the analyzer reflection ratio (*R*_*p*_/*R*) and a scaling factor for the recorded intensity (*F*, equivalent to *S*_0_) (Koide *et al.*, 1991 ▸; Schäfers *et al.*, 1999 ▸; Nahon & Alcaraz, 2004 ▸; Wang *et al.*, 2012 ▸). Selection of the input data files, entry of fitting parameters, and display of the results is made *via* a simple GUI shown in Fig. 3 ▸. The interface is designed to be user-friendly in order that large data sets may be processed quickly and easily.

The data from each rotation of the primary axis are stored in separate files which are read sequentially into the analysis software. Fitting can be carried out using the full data set, or using data from subsets of the angular motion from each rotation. In this way, any artifacts in the data which occur over certain angular ranges (caused by misalignment of the optics, sample imperfection, *etc*.) can be disregarded from the analysis. Spectra acquired at opposing positions of the secondary rotation (*e.g.* 0/180°, 45/225°, *etc*.) are equivalent, so these are averaged to eliminate the effect of angular mis­alignment between the rotation axes α and β. The software may also be used to generate the expected light intensity curves for a given set of fitting parameter values.

## 3. Multilayer optics   

Measurements at 375 eV were carried out using Cr/Sc multilayers optimized at this energy (number of periods *N* = 400, periodic thickness *d* = 2.57 nm, Cr/Sc thickness ratio Γ = 0.5) for both polarizing elements (Kimura *et al.*, 2005 ▸). This is within the so-called ‘water window’, between the *K* absorption edges of carbon and oxygen (284–540 eV) where soft X-rays are strongly absorbed by organic structures whereas water appears relatively transparent, making it important for imaging of biological specimens (Jiang & Chen, 2009 ▸; Carzaniga *et al.*, 2014 ▸; Rose *et al.*, 2015 ▸). Complete polarization analysis was used to determine the optimal phase retarder tilt angle θ_R_. As the magnitude of the coefficient to *P*_3_ is proportional to the phase shift parameter Δ, finding the tilt angle which maximizes this value reduces the error on the fitted *P*_3_ value.

**Figure 4:**
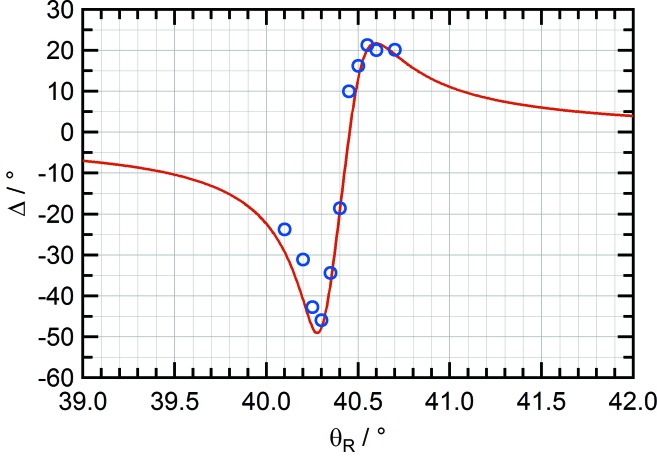
Comparison of the phase shift Δ induced by Cr/Sc transmission phase retarder determined by complete polarization analysis of linearly polarized light (blue circles) and theoretical calculation using IMD (red line).

Measurements of the phase shift at a variety of tilt angles are compared with the expected phase shift curve calculated using the *XOP* extension *IMD* (Windt, 1998 ▸) (see Fig. 4 ▸). The maximum phase shift at θ_R_ = 40.3° is found to be Δ = −45.9°, which is consistent with a simulated interface roughness of 4 Å. The *s*-component and *p*-component transmissions of the phase retarder at 375 eV are *T*_*s*_ = 0.25% and *T*_*p*_ = 0.8%, respectively. The analyser *s*-component reflectivity *R*_*s*_ is 26% and the *p*-component reflectivity *R*_*p*_ is 1.2%.

## 4. Polarization measurements   

**Figure 5:**
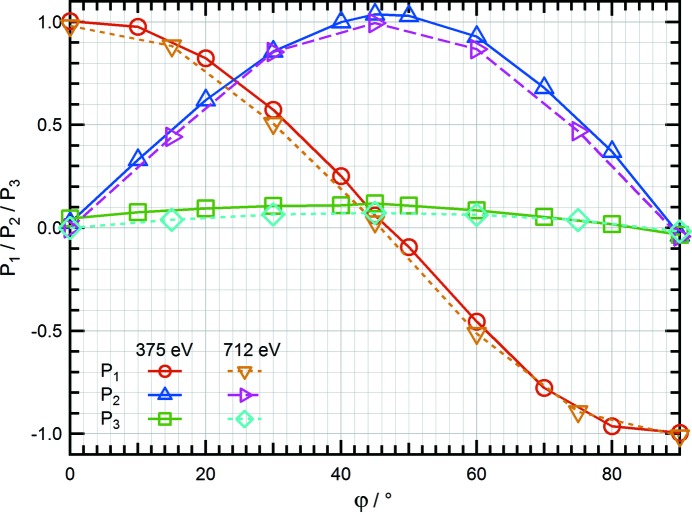
Comparison of Stokes–Poincaré polarization parameters obtained from measurements of the beam when the undulator is set to emit linearly polarized light at a range of angles φ between 0° (LH) and 90° (LV).

Polarimetry tests for light polarized at arbitrary linear angles were carried out using the downstream undulator on I06. The undulator was set to emit linearly polarized light at a range of arbitrary angles φ between 0° (LH) and 90° (LV). During this test the upstream undulator and phasing unit gaps were set to their maximum values so they had no influence on the electron beam. As shown in Fig. 5 ▸, we find that as the polarization angle increases from LH the purity of the linear polarization deteriorates slightly by the introduction of a small *P*_3_ component, with the maximum *P*_3_ of ∼0.1 being found at φ = 45°. As φ is further increased, the *P*_3_ contamination is reduced until a pure LV state is reached. Previously, the same measurement has been carried out on I06 at 712 eV using W/B_4_C multilayer optics (retarder: *N* = 320, *d* = 1.74 nm, Γ = 0.236, analyser: *N* = 250, *d* = 1.24 nm, Γ = 0.35) (Wang *et al.*, 2011 ▸). As shown in Fig. 5 ▸, the same behaviour was observed at 712 eV. An offset between the electron beam and the undulator can potentially cause the polarization contamination and further investigation will be performed to verify this. The overall uncertainty in each Stokes–Poincaré parameter is estimated to be ±1–2%. However, the obtained value of *P*_3_ is extremely sensitive to the accuracy with which the retarder phase shift is determined experimentally.

**Figure 6:**
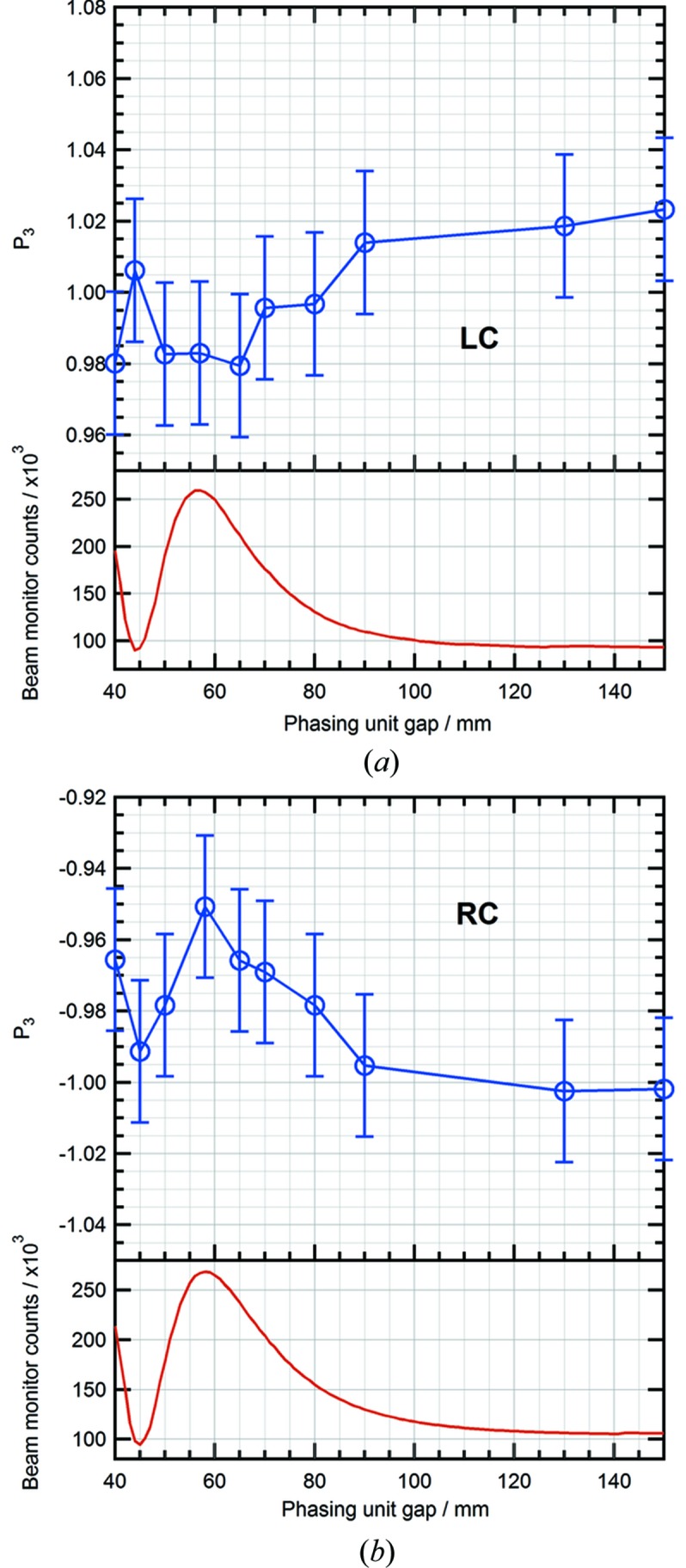
Variation of Stokes–Poincaré parameter *P*_3_ with phasing unit gap for (*a*) left and (*b*) right circular polarized light. The small difference in measured intensity of LC and RC light is due to a slight inaccuracy in the undulator calibration polynomials which determine the gaps and row phases for each energy and polarization state (easily accounted for in experiments because the photon flux monitor is used for normalization).

A further set of polarimetry measurements was carried out to investigate the influence of the phasing unit on the resultant X-ray beam polarization. In this case, both the upstream and downstream undulator configurations were synchronized to emit LC or RC light while the value of the PU gap was varied. The results shown in Fig. 6 ▸ clearly indicate the existence of a negative correlation between the total photon flux and the degree of circular polarization. For RC light, the degree of circular polarization is at its lowest when the PU gap is reduced to 58 mm, which coincides with the point at which peak photon flux is measured. Conversely, as the PU gap is further reduced, a minimum in the measured flux is seen with a corresponding maximum in the degree of circular polarization. Identical behaviour is also observed in the measurements of LC light. In theory, the flux will reach a maximum if the phasing unit is set to so that the phase difference between the centres of two adjacent undulators reaches an integer multiple of 2π (Diviacco *et al.*, 2011 ▸). Similarly to the above measurement for a single undulator, misalignment between the electron beam and the two undulators might also contribute to the deterioration of circular polarization case for the dual undulators. Even though the phasing unit is set for maximum flux, the degree of circular polarization will not be perfectly 100% due to such misalignment. However, the maximum of degree circular polarization can still be achieved if such an offset can be compensated by tuning the phasing unit. Although this assumption on the origin of the contamination will be further checked in forthcoming experiments, it is still important for beamline users to be aware of these characteristics when performing polarization-sensitive experiments.

## 5. Conclusion   

Complete polarization analysis with the high-precision Diamond soft X-ray polarimeter has been used to characterize the photon polarization for several undulator configurations on beamline I06 at Diamond Light Source. This includes the measurement of a photon beam produced by simultaneous operation of two undulator devices. In the measurements presented here we find a deterioration of the polarization purity when using linearly polarized light at arbitrary angles. A negative correlation is also seen between the degree of circular polarization and the photon flux when a phasing unit is used. We believe these precise polarization measurements provide valuable input for many synchrotron facilities where dual undulators are used for polarization-related measurements.
